# Oxidative Stress-Mediated Reperfusion Injury 2014

**DOI:** 10.1155/2015/689416

**Published:** 2015-07-22

**Authors:** Zhengyuan Xia, Yanfang Chen, Qian Fan, Mengzhou Xue, Ke-xuan Liu

**Affiliations:** ^1^Department of Anesthesiology, The University of Hong Kong, 102 Pokfulam Road, Hong Kong; ^2^Department of Anesthesiology, The Second Affiliated Hospital & Yuying Children's Hospital of Wenzhou Medical University, Wenzhou, Zhejiang 325027, China; ^3^Department of Pharmacology & Toxicology, Boonshoft School of Medicine, Wright State University, 3640 Colonel Glenn Highway, Dayton, OH 45435, USA; ^4^Cardiovascular Department, Guangzhou Institute of Cardiovascular Disease, The Second Affiliated Hospital of Guangzhou Medical University, Guangzhou 510000, China; ^5^Department of Cardiology, Beijing Anzhen Hospital, Capital Medical University, Beijing 100029, China; ^6^Department of Neurology, The First Affiliated Hospital, Henan University, 357 Ximen Street, Kaifeng 470010, China; ^7^Department of Anesthesiology, The First Affiliated Hospital, Sun Yat-Sen University, Guangzhou 510080, China

Ischemia/reperfusion injury (IRI) and organ failure especially IRI-induced remote and multiple organ failure contribute significantly to postoperative mortality and morbidity, and reperfusion induced oxidative stress plays a critical role in this pathology [1, 2].

Reactive oxygen species (ROS) induced vascular endothelial dysfunction plays an important role in the development of IRI in various organs. In this special issue, Q. Yang et al. reported that the otherwise cardiac protective polymerized hemoglobin, when used at high dose, failed to alleviate cardiac IRI due to induction of oxidative damage in coronary artery. On the other hand, natural herbal extracts such as Licochalcone B as reported by J. Han et al. in this special issue conferred protection against myocardial IRI through attenuating ischemia-reperfusion induced oxidative damage.

The intravenous anesthetic propofol possesses antioxidant capacity and has been shown to attenuate IRI in patients undergoing cardiac surgery and in animal models of myocardial [3] and intestinal IRI [4]. In this special issue, X. Gan et al. further identified that propofol attenuated intestinal IRI through inhibiting the ROS generating NADPH oxidase mediated mast cell activation. Given that mast cell activation has been shown to play an important role in IRI-induced remote organ injury [5], the finding by X. Gan et al. may promote further in-depth studies, both experimental and clinical, regarding the potential protective effects of propofol in attenuating or preventing postischemic remote organ injuries.

Disturbances of* mitochondrial homeostasis* play critical roles in Acute Organ Failure [6] and in postischemic cellular injury [7], while the governing mechanism of* mitochondrial homeostasis alterations during these pathologies is largely unclear*. In this special issue, S. Cao et al. provided genome-wide expression profiling of cardiomyocytes subjected to hypoxia-reoxygenation injury in an effort to uncover the roles of mitoKATP in energy homeostasis and its regulation.

We hope that the original and review articles presented in this special issue, representing the current advances in the oxidative stress-mediated ischemia-reperfusion injury, with respect to their potential impact in cellular survival pathways and therapeutic strategies, will stimulate further exploration of this important area. Despite diversity, it is our belief that the articles comprised in this special issue could represent an important advancement and contribution to improve our knowledge of the mechanisms governing reperfusion injury.
